# Abstracts from global literature

**Published:** 2010

**Authors:** Pankil Patel, Ravi Khambhati, Y. S. Marfatia

**Affiliations:** Department of Skin VD, Government Medical Collage and SSG Hospital, Vadodara, India

Gordon RJ, Chez N, Jia H, Zeller B, Sobieszczyk M, Brennan C, *et al*. The NOSE Study (Nasal Ointment for *Staphylococcus aureus* Eradication): A Randomized Controlled Trial of Monthly Mupirocin in HIV-Infected Individuals. J Acquir Immune Defic Syndr 2010;55:466-72.

Background:

HIV-positive patients at HELP/PSI, Inc, an in-patient drug rehabilitation center, had a high baseline prevalence of *Staphylococcus aureus* colonization (49%) and incidence of infection (17%) in a previous year-long study.

Materials and Methods:

A randomized, double-blinded, placebo-controlled study was conducted to determine whether repeated nasal application of mupirocin ointment would decrease the odds of *S. aureus* nasal colonization in 100 HELP/PSI patients over an 8-month period. A 5-day course of the study drug was given monthly, and colonization was assessed at baseline and 1 month after each treatment. *S. aureus* infection was a secondary outcome.

Results:

In repeated-measures analysis, mupirocin reduced the odds of monthly *S. aureus* nasal colonization by 83% compared with placebo [adjusted odds ratio (OR_adj_) = 0.17; *P* < 0.0001]. Subjects colonized at study entry had a 91% reduction in subsequent colonization (OR_adj_= 0.09; *P* < 0.0001). Mupirocin also suppressed *S. aureus* colonization in subjects not colonized at baseline (OR_adj_ = 0.23; *P* = 0.006). There was no difference in infection rates between the mupirocin and placebo groups (hazard ratio = 0.49, *P* = 0.29).

Conclusions:

Monthly application of nasal mupirocin significantly decreased *S. aureus* colonization in HIV patients in residential drug rehabilitation. Monthly mupirocin application has a potential role in long-term care settings or in HIV-positive patients with high rates of *S. aureus* colonization and infection.

