# Supplementary material for: Abstracts from global literature
Source: Indian J Sex Transm Dis AIDS. 2010 Jul-Dec;31(2):122–4. (PMC3122600)
Supplement: Related texts

# Abstract: Knowledge of human papillomavirus among high school students can be increased by an educational intervention

Gottvall MR, Tydén T, Höglund AT, Larsson M. Knowledge of human papillomavirus among high school students can be increased by an educational intervention. Int J STD AIDS 2010;21:558-62.

The aim of this study was to evaluate the effect of an educational intervention concerning human papillomavirus (HPV) directed at Swedish first year high school students. The intervention consisted of a class room lesson, a website, and a folder. Outcome variables were knowledge of HPV and attitudes to preventive methods, such as HPV vaccination, condom use, and Pap smear testing. An intervention group (n = 92) was matched with 2 comparison groups (n = 184). At baseline, the median score for HPV knowledge was 1 out of 10 in both the groups. At follow-up, the median knowledge score had increased to 6 in the intervention group, but was still 1 in the comparison group (*P* < 0.001). Attitudes to HPV vaccination, condom use, and Pap smear testing remained the same (*P* > 0.05). In conclusion, a short school-based intervention can greatly increase the students’ knowledge about HPV, but attitudes and behaviors are less easy to influence.

# Abstract: Risk factors for immune reconstitution inflammatory syndrome under combination antiretroviral therapy can be aetiology specific

Espinosa E, Ormsby CE, Vega-Barrientos RS, Ruiz-Cruz M, Moreno-Coutiño G, Peña-Jiménez A, *et al*. Risk factors for immune reconstitution inflammatory syndrome under combination antiretroviral therapy can be aetiology specific. Int J STD AIDS 2010;21:573-9.

In order to discriminate general from aetiology-specific risk factors for immune reconstitution inflammatory syndrome (IRIS), we followed up, during 6 months, 99 patients with advanced HIV infection commencing antiretroviral therapy (ART) without active opportunistic infections or evident inflammation. IRIS predictors were determined by univariate analysis using clinical data from 76 ART-responding patients either completing follow-up or developing IRIS, and by multivariate analysis of inflammation, disease progression, and nutrition status variables. We identified 23 primary IRIS events (30.3%). Univariate predictors for all IRIS events were higher platelet counts and lower CD4/CD8 ratio, whereas subclinical inflammation was the multivariate predictor. Platelets, alkaline phosphatase levels and %CD8 T cells in univariate analysis also predicted mycobacteria-associated IRIS independently, remaining elevated during follow-up. Herpes virus IRIS was predicted by platelets and inflammation. Indicators of advanced HIV disease and subclinical inflammation jointly predict IRIS, and some are specific of the underlying microbial aetiology, possibly explaining previous reports.

# Abstract: Problems with condoms may be reduced for men taking ample time to apply them

Crosby RA, Graham CA, Yarber WL, Sanders SA. Problems with condoms may be reduced for men taking ample time to apply them. Sex Health 2010;7:66-70.

Background:

One potentially important antecedent of experiencing problems with condom use during penile–vaginal sex is the amount of time that men (and perhaps women) allow for condom application. To examine whether men reporting that ample time was available to apply a male condom (the last time a condom was used for penile–vaginal sex) were also less likely to report problems with condom use, such as breakage, slippage, and erection difficulties, during that sexual event.

Materials and Methods:

A convenience sample of men (n = 440) was recruited via advertisements in newspapers (2 urban and 1 small town) and a blog on the website of a condom sales company. Men completed a questionnaire posted on the website of The Kinsey Institute for Research in Sex, Gender, and Reproduction. Inclusion criteria were that participants were at least 18 years old; used condoms for penile–vaginal intercourse in the past 3 months; and able to read English.

Results:

In controlled, event-specific analyses, men reporting that they did not have sufficient time for condom application were ~3 times more likely to report breakage and ~2.4 times more likely to report slippage. In addition, men who reported that they lacked time for condom application were ~2.4 times more likely to experience any of the 9 sexual problems, 3.4 times more likely to report difficulty with erection, 2.1 times more likely to report reduced sexual pleasure, 2.2 times more likely to report reduced sexual pleasure of their female partner, and 2.6 times more likely to report that the condom irritated their partner’s vagina.

Conclusions:

This is the first study using an event-specific analysis to examine the effect of not having enough time for condom application on condom breakage, slippage, and several outcomes related to sexual pleasure. Sexually transmissible infections and pregnancy prevention messages should include recommendations to men to take their time applying condoms.

# Abstract: Minimal impact of circumcision on HIV acquisition in men who have sex with men

Londish GJ, Templeton DJ, Regan DG, Kaldor JM, Murray JM. Minimal impact of circumcision on HIV acquisition in men who have sex with men. Sex Health 2010;7:463-70.

Background:

Men who have sex with men (MSM) are disproportionately affected by HIV. The proven efficacy of circumcision in reducing the risk of HIV acquisition among African heterosexual males has raised the question of whether this protective effect may extend to MSM populations. We examined the potential impact of circumcision on an HIV epidemic within a population of MSM.

Methods:

A mathematical model was developed to simulate HIV transmission in an MSM population. The model incorporated both circumcision and seropositioning, and was used to predict the reduction in HIV prevalence and incidence as a result of the 2 interventions. Estimates for the time required to achieve these gains were also calculated.

Results:

We derive simple formulae for the decrease in HIV prevalence with increased circumcision. Our model predicts that if an initially uncircumcised MSM population in a developed country with a baseline HIV prevalence of 10% underwent universal circumcision, HIV incidence would only be reduced to 95% of pre-intervention levels and HIV prevalence to 9.6% after 20 years. In the longer term, our model predicts that prevalence would decrease from 10% to 6% only, but this would take several generations to achieve. The effectiveness of circumcision increases marginally with higher degrees of seropositioning.

Conclusions:

The results of these calculations suggest that circumcision as a public health intervention will not produce a substantial decrease in HIV prevalence or incidence among MSM in the near future, and only modest reductions are achievable in the long term.

# Abstract: Association of oncogenic and nononcogenic human papillomavirus with HIV incidence

Auvert B, Lissouba P, Cutler E, Zarca K, Puren A, Taljaard D. Association of oncogenic and nononcogenic human papillomavirus with HIV incidence. J Acquir Immune Defic Syndr 2010;53:111-6.

Objective:

Little is known about the interaction between human papillomavirus (HPV) and HIV. This study aimed to explore the association of oncogenic (high risk) and nononcogenic (low risk) HPV with HIV incidence.

Materials and Methods:

We used 1683 urethral swabs collected at the last follow-up visit of a male circumcision trial conducted in Orange Farm (South Africa). Swabs analyses and HPV genotyping were performed by polymerase chain reaction. We estimated HIV adjusted incidence rate ratios (aIRRs) and 95% confidence intervals (CIs) using survival analysis. Background characteristics, male circumcision status, sexual behavior, HPV status, and other sexually transmitted infections were used as covariates.

Results:

The prevalence of HR and LR HPV was 14.0% (95% CI: 12.4–15.7) and 17.3% (95% CI: 15.6–19.2), respectively. When controlling for HR-HPV status, LR-HPV status was not associated with HIV incidence (aIRR = 1.13, 95% CI: 0.40–3.16; P = 0.82). When controlling for all covariates, HIV incidence increased significantly with HR-HPV positivity (aIRR = 3.76, 95% CI: 1.83–7.73, P < 0.001) and with the number of HR-HPV genotypes (adjusted-P linear trend = 0.0074).

Conclusions:

Several explanations could account for our findings. One is that HR-HPV facilitates HIV acquisition. The association of HPV with HIV acquisition requires further investigations.

# Abstract: Implementation science for the prevention and treatment of HIV/AIDS

Schackman BR. Implementation science for the prevention and treatment of HIV/AIDS. J Acquir Immune Defic Syndr 2010;55:S27-31.

Implementation science is the scientific study of methods to promote the integration of research findings and evidence-based interventions into health care policy and practice and hence to improve the quality and effectiveness of health services and care. Implementation science is distinguished from monitoring and evaluation by its emphasis on the use of the scientific method. The origins of implementation science include operations research, industrial engineering, and management science. Today, implementation science encompasses a broader range of methods and skills, including decision science and operations research, health systems research, health outcomes research, health and behavioral economics, epidemiology, statistics, organization and management science, finance, policy analysis, anthropology, sociology, and ethics. Examples of implementation science research are presented for HIV prevention (prevention of mother-to-child transmission of HIV, male circumcision) and HIV and drug use (syringe distribution, treating drug users with antiretroviral therapy and opioid substitution therapy). For implementation science to become an established field in HIV/AIDS research, there needs to be better coordination between funders of research and funders of program delivery and greater consensus on scientific research approaches and standards of evidence.

# Abstract: The carcinogenicity of smegma: Debunking a myth

Van Howe RS, Hodges FM. The carcinogenicity of smegma: Debunking a myth. J Eur Acad Dermatol Venereol 2006;20:1046-54

Background:

Smegma is widely believed to cause penile, cervical, and prostate cancer. This nearly ubiquitous myth continues to permeate the medical literature despite a lack of valid supportive evidence.

Materials and Methods:

A historical perspective of medical ideas pertaining to smegma is provided, and the original studies in both animals and humans are reanalyzed using the appropriate statistical methods.

Results:

Evidence supporting the role of smegma as a carcinogen is found wanting.

Conclusions:

Assertions that smegma is carcinogenic cannot be justified on scientific grounds.
